# Transcriptomic and Metabolomic Analysis of Liver Cirrhosis

**DOI:** 10.2174/1386207326666230717094936

**Published:** 2024-03-28

**Authors:** Xiao Kuang, Jinyu Li, Yiheng Xu, Lihong Yang, Xiaoxiao Liu, Jinhui Yang, Wenlin Tai

**Affiliations:** 1 Department of Clinical Laboratory, Yunnan Molecular Diagnostic Center, The Second Affiliated Hospital of Kunming Medical University, Kunming, China;; 2 Kunming Medical University, Kunming, China;; 3 Department ofGastroenterology, Yunnan Research for Liver Diseases, The Second Affiliated Hospital of Kunming Medical University, Kunming, China

**Keywords:** Bioinformatics, liver cirrhosis, metabolome, transcriptome, biomarkers, differentially expressed mRNAs, differentially expressed miRNAs

## Abstract

**Background:**

Liver cirrhosis is one of the leading causes of decreased life expectancy worldwide. However, the molecular mechanisms underlying liver cirrhosis remain unclear. In this study, we performed a comprehensive analysis using transcriptome and metabolome sequencing to explore the genes, pathways, and interactions associated with liver cirrhosis.

**Methods:**

We performed transcriptome and metabolome sequencing of blood samples from patients with cirrhosis and healthy controls (1:1 matched for sex and age). We validated the differentially expressed microRNA (miRNA) and mRNAs using real-time quantitative polymerase chain reaction.

**Results:**

For transcriptome analysis, we screened for differentially expressed miRNAs and mRNAs, analyzed mRNAs to identify possible core genes and pathways, and performed co-analysis of miRNA and mRNA sequencing results. In terms of the metabolome, we screened five pathways that were substantially enriched in the differential metabolites. Next, we identified the metabolites with the most pronounced differences among these five metabolic pathways. We performed receiver operating characteristic (ROC) curve analysis of these five metabolites to determine their diagnostic efficacy for cirrhosis. Finally, we explored possible links between the transcriptome and metabolome.

**Conclusion:**

Based on sequencing and bioinformatics, we identified miRNAs and genes that were differentially expressed in the blood of patients with liver cirrhosis. By exploring pathways and disease-specific networks, we identified unique biological mechanisms. In terms of metabolomes, we identified novel biomarkers and explored their diagnostic efficacy. We identified possible common pathways in the transcriptome and metabolome that could serve as candidates for further studies.

## 1 INTRODUCTION

Liver cirrhosis is the clinical outcome of many chronic liver diseases. Over the past 30 years, the number of liver cirrhosis-related deaths has steadily increased worldwide. The global death toll from cirrhosis increased from 676,000 in 1980 (1.54% of global deaths) to more than one million in 2010 (approximately 2% of all deaths that year). Liver cirrhosis can occur in people of any age or sex and has become a major cause of the global medical burden [1, 2]. Nevertheless, the specific number of patients with liver cirrhosis cannot be clarified at present because 73% of patients with cirrhosis-related deaths were admitted to the hospital for the first time during the decompensated period of liver cirrhosis, and there is a lack of early diagnosis and intervention treatment methods [2]. Therefore, it is particularly important to identify new molecular markers of liver cirrhosis to help diagnose and study the pathogenesis of liver cirrhosis and identify new treatments.

With the development of high-throughput omics technologies for different tissue levels, it is possible to conduct systematic and relevant multi-omics analyses of the pathogenesis of diseases [3]. Transcriptomics involves second-generation RNA sequencing. Because RNA sequencing provides single-base resolution on a genome-wide scale, it greatly improves our opportunities for quantitative and qualitative aspects of the human transcriptome [4]. Using bioinformatics tools, many potential biomarkers of related diseases can be identified using RNA sequencing [5]. Metabolomics is the quantitative study of all low-molecular-weight metabolites in an organism or biological sample in a unit of time under specific environmental conditions. Low-molecular-weight metabolites include peptides, alkaloids, nucleic acids, amino acids, and organic acids [6]. Metabolomics is recognized as a new and powerful technique for biomarker discovery and the exploration of dynamic areas, especially information that differentiates diseased and non-diseased states [7].

In this study, we screened for differentially expressed genes using microRNA (miRNA) and mRNA sequencing of blood samples from cirrhotic patients and healthy controls (1:1 matched for sex and age) and analyzed them using bioinformatics tools. Pathways and key biomarkers associated with cirrhosis were identified, and their relationship with the disease was explored. In addition, differential metabolites were screened by metabolomics in the plasma of cirrhotic patients and healthy controls (1:1 matched for sex and age). And we performed a joint analysis of transcriptome results and metabolome results.

## 2 MATERIALS AND METHODS

### 2.1 Recruitment of Study Participants

This study complied with the ethical guidelines of the Declaration of Helsinki and was approved by the Ethics Committee of the Second Affiliated Hospital of Kunming Medical University (ethics approval document number: PJ-2020-25). Written informed consent was obtained from all recruited patients. Six participants underwent transcriptome sequencing, 52 underwent metabolome sequencing, and 14 underwent real-time fluorescence quantitative polymerase chain reaction (RT-qPCR) validation.

### 2.2 Sequencing of miRNAs and Data Analysis Methods

The final ligation PCR products were sequenced on the BGISEQ-500 platform (BGI-Shenzhen, China). Small RNA expression levels were calculated by counting the absolute number of molecules using unique molecular identifiers [8]. Differential expression analysis was performed using DEGseq [9], Q value ≤0.001, and the absolute value of Log2Ratio ≥1 as the default threshold to judge the significance of expression differences. To annotate gene functions, all target genes were aligned against the Kyoto Encyclopedia of Genes (KEGG) and Gene Ontology (GO) databases. GO and KEGG enrichment analyses of target genes were performed using phyper, a function of R. The *P*-value was corrected using the Bonferroni method, and a corrected *P*-value ≤0.05 was set as a threshold. GO or KEGG terms fulfilling this condition were defined as significantly enriched terms.

### 2.3 Sequencing of mRNAs and Data Analysis Methods

The final mRNA library was amplified with phi29 (Thermo Fisher Scientific, MA, USA) into DNA nanoballs (DNBs), where one molecule had more than 300 copies. The DNBs were loaded into patterned nanoarrays to generate reads of 100 bases on the opposite end of the BGISEQ500 platform (BGI-Shenzhen, China). A heatmap was prepared using heatmap (v1.0.8) according to the gene expression in various samples. Differential expression analysis was performed using DESeq2 (v1.4.5) [10] with a Q-value ≤0.05. To gain insight into the phenotypic changes, GO (http://www.geneontology.org/) and KEGG (https://www.kegg.jp/) enrichment analyses of differentially expressed annotated genes were performed using Phyper (https://en.wikipedia.org/wiki/Hypergeometric_distribution) based on the hypergeometric test. The significance levels of terms and pathways were corrected by the Q value with a rigorous threshold (Q value ≤0.05) by Bonferroni correction [11].

### 2.4 RT-qPCR

Primer Express software (version 5.0, Applied Biosystems) was used to design miRNA and mRNA primers, which were synthesized by Tsingke Biotechnology Co. miRNA was analyzed using miRNA RT Enzyme MixTaqMan (TIANGEN, China), and mRNA was analyzed using Servicebio RT Enzyme Mix (Servicebio, China) for reverse transcription of total RNA extracted from blood to cDNA. RT-qPCR was performed at a volume of 20 µL per reaction. miRNA was analyzed using miRcute Plus miRNA PreMix (TIANGEN, China), mRNA was fluorescently stained using SYBR Green qPCR Master Mix (Bimake, USA), and both were amplified and analyzed using an ABI 7500 Real-Time PCR system (Applied Biosystems, Foster City, CA, USA). The U6 expression level was used as an endogenous control for miRNA, and the GAPDH expression level was used as an endogenous control for mRNA for standardization. All assays were performed in triplicate.

### 2.5 LC-MS/MS and Metabolome Analysis of Plasma

This experiment was performed at the Institute of Life Sciences, BGI-Shenzhen, China, using a Waters 2D UPLC (Waters, USA) tandem with a Q Exactive high-resolution mass spectrometer (Thermo Fisher Scientific, USA) for metabolite separation and detection. To provide more reliable experimental results during instrument testing, samples were randomly arranged to reduce systematic errors. One QC sample was interspersed every 10 samples. Raw data were processed using Progenesis QI 2.0 data analysis software (Nonlinear Dynamics, Newcastle, UK) for peak picking, alignment, and normalization to produce peak intensities for retention time and m/z data pairs. Further statistical analysis was performed on the resulting normalized peak intensities using the in-house developed software metaX (http://www.bioconductor.org/packages/devel/bioc/html/metaX.html). In metaX processing, metabolic features detected in <50% of QC samples or <80% of experimental samples were removed. In addition, all metabolic features with a coefficient of variation (CV) of >30%, as calculated for the QC samples, were also removed. The identification of metabolites was a combined result of the BGI self-built standard library, mzCloud, and ChemSpider (HMDB, KEGG, LipidMaps) databases. Pathway analysis was performed using the MetaboAnalyst pathway tool (http://www.metaboanalyst.ca).

### 2.6 Statistical Analysis

The constructed receiver operating characteristic (ROC) curves were used to predict biomarkers in patients with cirrhosis. The RT-qPCR results were compared between the two groups using the unpaired t-test. Bioinformatics analysis of the sequencing data was performed using the Dr. Tom System (BGI, Shenzhen, China). GraphPad Prism (version 8.0) was used for data analyses. All two-sided *p* values <0.05 were considered statistically significant.

## 3 RESULTS

### 3.1 Transcriptome Group Clinical Characteristics Data

In this study, we screened for differentially expressed genes by performing miRNA and mRNA sequencing in cirrhotic patients and healthy controls (1:1 matched for sex and age). The cirrhosis and control groups consisted of two men and one woman, respectively. The mean age of the patients in both groups was 56. In the cirrhosis group, two patients had hepatitis B cirrhosis and one had autoimmune cirrhosis. There were no statistically significant differences in ALT levels between the two groups (cirrhosis group, 54.33 ± 19.13 *vs.* control group, 23.67 ± 8.21, *p* = 0.21). However, there was a statistically significant difference between the two groups in terms of AST levels (cirrhosis group, 59.00 ± 8.72 *vs.* control group, 20.00 ± 2.08, *p* = 0.01).

### 3.2 Differentially Expressed mRNAs in Liver Cirrhosis

**Fig. (1):**
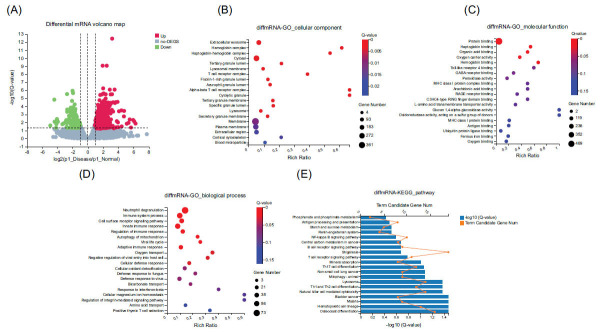
Differential mRNAs and their GO and KEGG analysis: (**A)** Volcano map of differential mRNAs; (**B)** Bubble plots of differentially expressed mRNAs at the cellular component level in GO functional analysis; (**C)** Bubble plots of differentially expressed mRNAs at the molecular functional level in GO functional analysis; (**D)** Bubble plots of differentially expressed mRNAs at the biological process level in GO functional analysis; (**E)** Plot of differentially expressed mRNAs in the KEGG pathway analysis.

We screened 732 differentially expressed mRNAs, of which 518 were upregulated and 214 were downregulated (Fig. **1A**). The differentially expressed mRNAs were mainly enriched in the cytoplasm and exosomes at the cellular component level in the GO functional analysis (Fig. **1B**). At the molecular function level, it was mainly enriched in protein binding, haptoglobin, organic acid binding, and hemoglobin activity (Fig. **1C**). At the biological process level, it was mainly enriched in neutrophil degranulation, immune system processes, and cell surface receptor signaling pathways (Fig. **1D**). The KEGG pathway analysis of differentially expressed mRNAs showed that seven pathways were significantly enriched, including osteoclast differentiation, hematopoietic cell lineage, and Th1 and Th2 cell differentiation (Fig. **1E**).

**Fig. (2):**
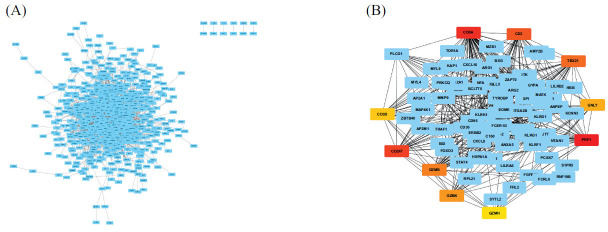
(**A**) The differentially expressed mRNAs were imported into the STRING database for annotation and analysis, and the protein interaction network map was obtained; (**B**) The protein interaction network was introduced into cytoscape for the screening of hub genes.

Differentially expressed mRNAs were imported into the STRING database for annotation and analysis to obtain a protein interaction network map (Fig. **2A**). Then the protein interaction network was imported into Cytoscape to screen out the core genes (CD8A, CD247, and PRF1; Fig. **2B**).

### 3.3 Combined Analysis of Differentially Expressed miRNAs and Differentially Expressed mRNAs

We intersected the predicted target genes of differentially expressed miRNAs with our sequenced differentially expressed mRNAs and found 117 identical genes. These 117 same mRNAs corresponded to the 17 differentially expressed miRNAs we sequenced. We then screened the corresponding miRNAs and mRNAs based on the principle that miRNAs and corresponding mRNAs are expressed at opposite levels and obtained the corresponding 14 miRNAs (hsa-miR-1271-5p, hsa-miR-150-5p, hsa-miR-152-3p, hsa-miR-188-5p, hsa-miR-195-5p, hsa-miR-24-3p, hsa-miR-301b-3p, hsa-miR-30a-5p, hsa-miR-376a-3p, hsa-miR-487a-3p, hsa-miR-615-3p, hsa-miR-7-5p, hsa-miR-708-5p, and hsa-miR-9-5p). The corresponding mRNAs are shown in Fig. (**3**).

### 3.4 RT-qPCR Validation of miRNAs and mRNAs

We selected three pairs of these 14 miRNAs and their corresponding mRNAs for RT-qPCR. RNA was extracted from the peripheral blood of cirrhotic patients and healthy controls (six cirrhotic patients *vs.* eight healthy controls). Both miRNAs and mRNAs detected in the two sample groups were statistically different, demonstrating the reliability of our transcriptome sequencing data (Fig. **4**).

### 3.5 Metabolome Group Clinical Characteristic Data

In this study, we screened for differential metabolites by metabolite sequencing of plasma from patients with cirrhosis and healthy controls (sex and age 1:1 matched). In terms of clinical characteristics, the two groups exhibited significant differences in alanine aminotransferase, aspartate aminotransferase, red blood cell count, hemoglobin, and platelet count but no differences in sex, age, and white blood cell count (Table **1**). Among them, patients with liver cirrhosis had much higher alanine aminotransferase and aspartate aminotransferase than the control group. However, the red blood cell count, hemoglobin and platelet count of cirrhotic patients were lower than those of normal controls.

### 3.6 Differential Metabolite Screening and Correlation Analysis

We used LC-MS/MS technology for untargeted metabolomic analysis, collecting data for both positive and negative ions to improve the metabolite coverage. In the positive ion mode, 1,548 differential metabolites were screened, of which 1,036 were upregulated, and 512 were downregulated. In the negative ion mode, 937 differential metabolites were screened, of which 630 were upregulated, and 307 were downregulated. Metabolic pathway enrichment analysis of differential metabolites based on the KEGG database showed that there were 16 main enriched pathways for differential metabolites. Five of them had significant differences in positive and negative ion modes: caffeine metabolism, metabolic pathways, cholesterol metabolism, primary bile acid biosynthesis, and bile secretion (Fig. **5**). We then analyzed the metabolites in these five metabolic pathways and selected the five metabolites with the most significant differences in expression: glycine chenodeoxycholate, d-urobilin, theophylline, 1-methylxanthine, and (2r,3s)-3-hydroxy-8-methyl-8-azabicyclo[3.2.^1^]octane-2-carboxylate methyl ester. Finally, we produced ROC curves for these five metabolites based on their relative concentrations to determine their diagnostic efficacy for liver cirrhosis. Among them, the best diagnostic results were for glycine chenodeoxycholate (area under the ROC curve, 0.9645; specificity, 88.46%; sensitivity, 100%), (2r,3s)-3-hydroxy-8-methyl-8-azabicyclo[3.2.^1^]octane-2-carboxylate methyl ester (area under the ROC curve, 0.8802; specificity, 73.08%; sensitivity, 88.46%), and D-urobilin (area under the ROC curve, 0.8210; specificity, 73.08%; and sensitivity, 88.46%; Table **2**).

In summary, we screened 732 differentially expressed mRNAs, of which 518 were upregulated, and 214 were downregulated. The differentially expressed mRNAs were mainly enriched in the cytoplasm and exosomes at the cellular component level in the GO functional analysis. At the molecular function level, it was mainly enriched in protein binding, haptoglobin, organic acid binding, and hemoglobin activity. At the biological process level, it was mainly enriched in neutrophil degranulation, immune system processes, and cell surface receptor signaling pathways. The KEGG pathway analysis of differentially expressed mRNAs showed that seven pathways were significantly enriched: including osteoclast differentiation, hematopoietic cell lineage, and Th1 and Th2 cell differentiation. Differentially expressed mRNAs were imported into the STRING database for annotation and analysis to obtain a protein interaction network map. The protein interaction network was then imported into Cytoscape to screen out the core genes (CD8A, CD247, and PRF1).

Then we performed untargeted metabolomics analysis using LC-MS/MS technology. Metabolic pathway enrichment analysis of differential metabolites based on the KEGG database showed that there were 16 main enriched pathways for differential metabolites. Five of them had significant differences in positive and negative ion modes: caffeine metabolism, metabolic pathways, cholesterol metabolism, primary bile acid biosynthesis, and bile secretion. We then analyzed the metabolites in these five metabolic pathways and selected the five metabolites with the most significant differences in expression: glycine chenodeoxycholate, d-urobilin, theophylline, 1-methylxanthine, and (2r,3s)-3-hydroxy-8-methyl-8-azabicyclo[3.2.^1^]octane-2-carboxylate methyl ester.

## 4 DISCUSSION

Liver cirrhosis is one of the leading causes of global lifespan reduction, although there are differences between countries with different incomes [12]. In fact, the incidence of cirrhosis may be higher than reported because initially compensated cirrhosis is often asymptomatic and undiagnosed [13]. The molecular mechanisms of liver cirrhosis are still unclear, and methods for its diagnosis and treatment are lacking.

In this study, we collected peripheral blood samples from patients with liver cirrhosis and healthy controls and then explored issues related to liver cirrhosis by performing a comprehensive transcriptomic and metabolomic analysis. At the transcriptional level, our analysis of differential mRNAs showed that they were mainly located in the cytoplasm and exosomes, and their molecular functions mainly in protein binding and protein activity. Their biological processes were mainly found in the immune system and cell surface receptor signaling pathways. We reviewed the relevant literature and found that exosomes are small vesicles in mature mammalian reticulocytes that produce specific plasma membrane proteins [14]. Studies show that exosomes transmit information between cells without direct cell-to-cell contact [15]. A study showed that exosome-derived MiR-214 targets CCN2 directly regulates the expression of CCN2, and inhibits the process of liver fibrosis [16]. Systemic immune dysfunction and abnormal liver autoimmunity were found in patients with liver cirrhosis [17]. This suggests that both exosomes and the immune system play important roles in liver cirrhosis. Thus, combined with the analysis of our study, we can draw this scientific hypothesis. Key genes that contribute to cirrhosis are concentrated in exosomes and promote the development of cirrhosis by regulating the binding and activity of key proteins through immune processes and cell surface receptor signaling.

The KEGG pathway analysis of differentially expressed mRNAs showed that seven pathways were significantly enriched, including osteoclast differentiation, hematopoietic cell lineage, and Th1 and Th2 cell differentiation (Fig. **1**). Related studies have shown that osteoporosis is one of the most serious complications of primary biliary cirrhosis (PBC), leading to an increased risk of fractures [18]. Studies have indicated that reduced bone formation may be one of the main causes of osteoporosis in PBC [19]. However, the specific mechanism is unclear. Based on our results, we speculated that hyperactive osteoclast differentiation leads to increased osteoclastogenesis, resulting in diminished bone formation in patients with cirrhosis. Focusing on genes in osteoclast differentiation pathways may provide a new direction for the treatment of osteoporotic complications in patients with liver cirrhosis.

Cytopenia is also a common complication in patients with liver cirrhosis. It is closely related to bleeding and infection in the decompensated phase of cirrhosis, and these factors accelerate disease progression [20]. Bone marrow endothelial cells can regulate hematopoiesis and are associated with various cytokines and receptors [21]. In our study, the pathway analysis also showed enrichment in the hematopoietic cell lineage and in Th1 and Th2 cell differentiation. Therefore, we hypothesize that imbalance in the immune microenvironment leads to abnormalities in the hematopoietic cell lineage and causes the development of cirrhosis. Our findings provide a reference and new direction for cellular and molecular experiments in liver cirrhosis.

In addition, we constructed a protein interaction network for the differentially expressed mRNAs to screen for core genes. The hub genes obtained included CD8A, CD247, and PRF1 (Fig. **2**). The intensity and number of immunosuppressive molecules were positively correlated with the severity of CD8+ T cell exhaustion, which resulted in diminished tumor suppressor effects [22]. Some data have shown that immunosuppression of the tumor microenvironment is related to the downregulation of CD247. CD247 downregulation also occurs in autoimmune diseases and inflammation with various etiologies and physiology. This suggests that this phenomenon has a common feature, and the occurrence of autoimmune diseases and tumors is related to chronic inflammation [23]. Cirrhosis begins with chronic inflammation that leads to abnormal repair and development. Therefore, we suggest that CD8A and CD247 may promote the progression of cirrhosis to HCC by mediating the chronic inflammatory response. PRF1 has been reported to be an anti-fibrotic gene [24]. Li *et al.* [25] found that PRF1 may be involved in the pathogenesis and progression of PBC. PRF1 may be a target for further exploration in the treatment of PBC.

**Fig. (3):**
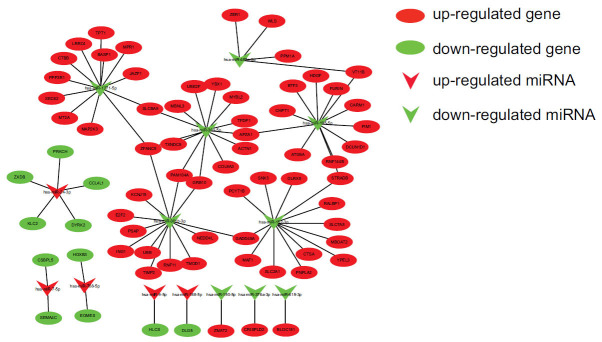
We screened the corresponding targets of miRNAs and mRNAs with opposite expression levels according to the sequencing expression levels, and obtained the corresponding 14 miRNAs. The 14 miRNAs screened out, their corresponding mRNAs, and their regulatory relationship.

**Fig. (4):**
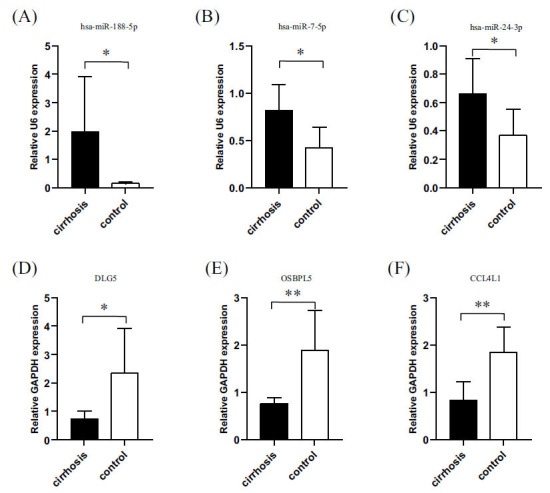
Validation of differentially expressed miRNAs and mRNAs in sequencing: (**A**) Hsa-miR-188-5p was highly expressed in the peripheral blood of cirrhotic patients and was statistically significant; (**B**) Hsa-miR-7-5p was highly expressed in the peripheral blood of cirrhotic patients and was statistically significant; (**C**) Hsa-miR-24-3p was highly expressed in the peripheral blood of cirrhotic patients and was statistically significant; (**D**) DLG5 was low and statistically significant in the peripheral blood of cirrhotic patients; (**E**) OSBPL5 was low and statistically significant in the peripheral blood of cirrhotic patients; (**F**) CCL4L1 was low and statistically significant in the peripheral blood of cirrhotic patients. (*p<0.05, **p<0.01).

Furthermore, we intersected the predicted genes of the differentially expressed miRNAs with the differentially expressed mRNAs obtained from our sequencing and found 117 identical genes. These 117 mRNAs corresponded to the 17 differentially expressed miRNAs we sequenced. We then screened the corresponding miRNAs and mRNAs based on the principle that miRNAs and corresponding mRNAs are expressed at opposite levels and obtained 14 miRNAs (Figure **3**). Three pairs of 14 miRNAs and their corresponding mRNAs were selected for RT-qPCR. Three miRNAs (hsa-miR-188-5p, hsa-miR-7-5p, and hsa-miR-24-3p), which have not yet been reported in liver cirrhosis, were verified by RT-qPCR.

Hsa-miR-188-5p is highly expressed in cancer and can be used as a prognostic indicator in HCC [26, 27]. Our results showed that hsa-miR-188-5p expression was remarkably elevated in cirrhosis. Perhaps hsa-miR-188-5p could be used as a predictor of the progression of cirrhosis to HCC. Hsa-miR-7-5p directly inhibits Bad protein involvement in immune regulation by improving T-lymphocyte apoptosis [28]. Thus, hsa-miR-7-5p may contribute to the inflammatory imbalance of cirrhosis by enhancing the immune response of the body by increasing the number of T lymphocytes. Therefore, we believe that hsa-miR-7-5p could be a target for studying inflammatory imbalance in cirrhosis. Hsa-miR-24-3p substantially inhibits non-small cell lung cancer (NSCLC) cell viability, migration, and tumor growth, while promoting apoptosis in NSCLC cells [29]. Since there are few studies on the relationship between hsa-miR-24-3p and cirrhosis, we suggest more research on whether it can inhibit the progression of cirrhosis to HCC.

A review of the relevant literature revealed that the three target genes (DLG5, OSBPL5, and CCL4L1) validated by RT-qPCR were sequentially associated with HCC, lipid metabolism, and immunity. Wang *et al.* [30] reported that DLG5 was downregulated in HCC and that reduced DLG5 expression was associated with poor survival in patients with HCC. Regarding OSBPL5, it was shown that the OSBPL family is mainly involved in lipid metabolism and related signal transduction [31]. Some studies have reported that CCL4L1 activates the signaling pathway of T cells by inducing NF-κB, creating an immune response effect that may play a vital role in the process of hepatitis B virus infection [32]. These studies reveal a possible relationship between cirrhosis and HCC with disorders of lipid metabolism and immune dysregulation. It also provides optional targets and key genes for subsequent cellular and molecular experiments, as well as for animal experiments.

**Fig. (5):**
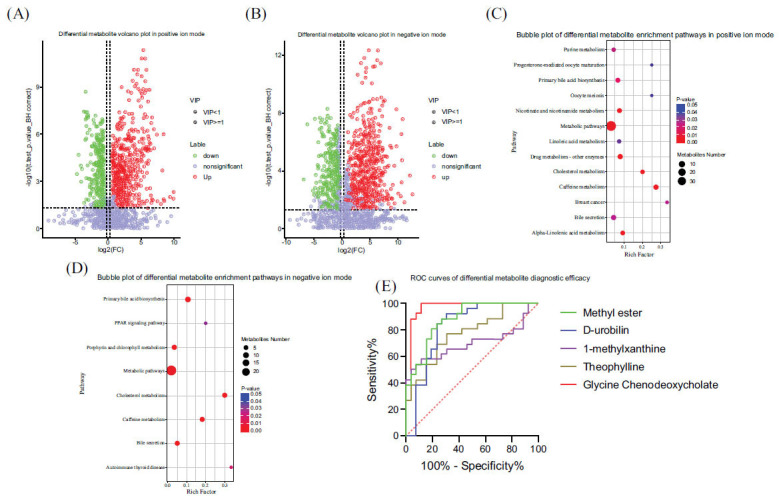
(**A** and **B**) Volcano plot of differential metabolites in positive and negative ion mode; (**C-D**) Bubble plot of differential metabolite enrichment in the KEGG pathway in positive and negative ion mode; (**E**) ROC curves of the diagnostic efficacy of five differential metabolites.

At the metabolome level, we screened for differential metabolites in the plasma of cirrhotic patients and healthy controls and analyzed these differential metabolites using the KEGG pathway. The enrichment analysis of differential metabolite metabolic pathways based on the KEGG database showed that differential metabolites were mainly enriched in 16 pathways, of which 5 had significant differences in positive and negative ion modes: caffeine metabolism, metabolic pathways, cholesterol metabolism, primary bile acid biosynthesis, and bile secretion (Fig. **5**). As the most important organ involved in the metabolism of substances in the body, pathological changes in the liver will inevitably lead to corresponding changes in the metabolic pathways associated with it and affect endogenous small-molecule metabolites [33]. Yang *et al.* [34] found that metabolite testing of urine distinguished patients with cirrhosis from those with HCC. And a biliary metabolomics study conducted by Nagana *et al.* [35] found that HCC can be distinguished from non-malignant liver disease based on bile acid and phospholipid levels in bile.

Therefore, we consider differentially expressed metabolites to be valuable biomarkers. We then analyzed the metabolites in these five metabolic pathways and selected the five metabolites with the most pronounced differences in expression: glycine chenodeoxycholate, d-urobilin, theophylline, 1-methylxanthine, and (2r,3s)-3-hydroxy-8-methyl-8-azabicyclo[3.2.^1^]octane-2-carboxylate methyl ester. One study found that the accumulation of glycine chenodeoxycholate in the liver is considered a crucial factor that causes liver damage [36]. Therefore, glycine chenodeoxycholate may serve as a sensitive diagnostic indicator or a new therapeutic target. In clinical practice, testing for urobilin can be effective in determining the cause of jaundice. We speculate that the D-urobilin in our study may have a similar role in aiding diagnosis.

Finally, we performed ROC curves based on the relative concentrations of these five metabolites to determine their diagnostic efficacy in liver cirrhosis. Among them, the best diagnostic results were for glycine chenodeoxycholate, (2r,3s)-3-hydroxy-8-methyl-8-azabicyclo[3.2.^1^]octane-2-car-boxylate methyl ester, and D-urobilin (Table **2**). This suggests that these three metabolites may serve as new non-invasive molecular markers in the diagnosis of cirrhosis.

The results of our transcriptome analysis found a major role for OSBPL5 in cholesterol metabolism, while the results of the metabolome also revealed enrichment of differential metabolites in the cholesterol metabolic pathway. Therefore, we hypothesized that in cirrhosis, hsa-miR-7-5p targets OSBPL5 to regulate cholesterol metabolism, which affects downstream metabolite changes. Wang *et al.* [37] found that in animals, the process of nonalcoholic fatty liver disease could be improved by regulating the metabolism of cholesterol and bile acids. Further, cholesterol metabolism has been documented as a potential therapeutic target in Duchenne muscular dystrophy [38]. For hsa-miR-7-5p, Li *et al.* [39] found that hsa-miR-7-5p inhibits glioma development by inhibiting NOVA2. Deng *et al.* [28] found that hsa-miR-7-5p directly inhibits Bad protein and participates in improving T-lymphocyte apoptosis. However, hsa-miR-7-5p and cholesterol metabolic pathways have not been reported in cirrhosis-related studies. Therefore, we hypothesized that hsa-miR-7-5p is involved in the regulation of cholesterol metabolism through OSBPL5 to influence the development of cirrhosis, which is a new research point.

The present study has several features. First, we executed rigorous sex- and age-matching of the two sample groups to exclude confounding factors as much as possible. Second, we performed sequencing and analysis of both the transcriptome and the metabolome. Third, depending on the origin of the cirrhosis group samples, our results may be more applicable to post-HBV cirrhosis and cirrhosis due to PBC. Fourth, we performed a joint analysis of transcriptomic and metabolomic results. Fifth, most patients in our cirrhotic group had ALT and AST levels no more than twice their upper limit of normal.

However, this study had some limitations. First, owing to the limitations of current research and technology, the current bioinformatics network used for sequencing data analysis is not complete and may also contain some false positive data. Second, the total sample size of our study was slightly small, and the results may have limitations.

## CONCLUSION

In conclusion, we elucidated the transcriptomic and metabolomic features of cirrhosis and discussed key genes and pathways. Based on sequencing and bioinformatics, we identified miRNAs and genes that are differentially expressed in the blood of cirrhotic patients. By exploring pathways and disease-specific networks, we identified unique biological mechanisms. In addition, we demonstrated novel metabolic biomarkers and explored their diagnostic efficacy. The common pathways of transcriptome and metabolome can be further investigated to provide new ideas for the pathogenesis and treatment of cirrhosis.

## Figures and Tables

**Table 1:** Clinical characteristics of the metabolome group.

**-**	**Cirrhosis Group (n=26)**	**Control Group (n=26)**	***p*-value**
Gender	-	-	-
Male n (%)	13(50)	13(50)	-
Female n (%)	13(50)	13(50)	-
Classification	-	-	-
HBV-hepatic cirrhosis	14(53.85)	-	-
HCV-hepatic cirrhosis	4(15.38)	-	-
PBC	8(30.77)	-	-
Age	51.92±1.81	51.92±1.81	>0.9999
ALT (U/L)	68.27±13.53	21.85±1.61	0.0013
AST (U/L)	65.88±10.37	19.35±0.59	<0.0001
RBC	3.78±0.17	4.99±0.09	<0.0001
Hb	117.90±4.23	152.70±3.43	<0.0001
PLT	112.10±20.44	237.20±11.07	<0.0001
WBC	4.50±0.60	6.43±0.32	0.0066

**Abbreviations:** HBV: Hepatitis B Virus; HCV: Hepatitis C Virus; ALT: Alanine Aminotransferase; AST: Aspartate Aminotransferase; RBC: Red Blood Count; Hb: Hemoglobin; PLT: Platelet; WBC: White Blood Cell Count.

**Table 2:** Diagnostic efficacy of different metabolites.

**Diff-metabolites**	**Sensitivity**	**Specificity**	**AUC**
Gly	100%	88.46%	0.9645
Theo	76.92%	69.23%	0.7707
1-methy	53.85%	92.31%	0.6982
Duro	88.46%	73.08%	0.8210
Methyl	88.46%	73.08%	0.8802

**Abbreviations:** Diff-metabolites: Differential metabolites; Gly: Glycine chenodeoxycholate; Theo: Theophylline; 1-methy:1-methylxanthine; Duro: D-urobilin; methyl:(2r,3s)-3-hydroxy-8-methyl-8-azabicyclo[3.2.1]octane-2-carboxylate methyl ester.

## Data Availability

The data sets used and/or analyzed during the current study are available from the corresponding author [W.T.] upon reasonable request.

## LIST OF ABBREVIATIONS

GO: Gene Ontology
KEGG: Kyoto Encyclopedia of Genes
miRNA: microRNA
ROC: Receiver Operating Characteristic

## References

[1] Lozano R., Naghavi M., Foreman K., Lim S., Shibuya K., Aboyans V., Abraham J., Adair T., Aggarwal R., Ahn S.Y., AlMazroa M.A., Alvarado M., Anderson H.R., Anderson L.M., Andrews K.G., Atkinson C., Baddour L.M., Barker-Collo S., Bartels D.H., Bell M.L., Benjamin E.J., Bennett D., Bhalla K., Bikbov B., Abdulhak A.B., Birbeck G., Blyth F., Bolliger I., Boufous S., Bucello C., Burch M., Burney P., Carapetis J., Chen H., Chou D., Chugh S.S., Coffeng L.E., Colan S.D., Colquhoun S., Colson K.E., Condon J., Connor M.D., Cooper L.T., Corriere M., Cortinovis M., de Vaccaro K.C., Couser W., Cowie B.C., Criqui M.H., Cross M., Dabhadkar K.C., Dahodwala N., De Leo D., Degenhardt L., Delossantos A., Denenberg J., Des Jarlais D.C., Dharmaratne S.D., Dorsey E.R., Driscoll T., Duber H., Ebel B., Erwin P.J., Espindola P., Ezzati M., Feigin V., Flaxman A.D., Forouzanfar M.H., Fowkes F.G.R., Franklin R., Fransen M., Freeman M.K., Gabriel S.E., Gakidou E., Gaspari F., Gillum R.F., Gonzalez-Medina D., Halasa Y.A., Haring D., Harrison J.E., Havmoeller R., Hay R.J., Hoen B., Hotez P.J., Hoy D., Jacobsen K.H., James S.L., Jasrasaria R., Jayaraman S., Johns N., Karthikeyan G., Kassebaum N., Keren A., Khoo J-P., Knowlton L.M., Kobusingye O., Koranteng A., Krishnamurthi R., Lipnick M., Lipshultz S.E., Ohno S.L., Mabweijano J., MacIntyre M.F., Mallinger L., March L., Marks G.B., Marks R., Matsumori A., Matzopoulos R., Mayosi B.M., McAnulty J.H., McDermott M.M., McGrath J., Memish Z.A., Mensah G.A., Merriman T.R., Michaud C., Miller M., Miller T.R., Mock C., Mocumbi A.O., Mokdad A.A., Moran A., Mulholland K., Nair M.N., Naldi L., Narayan K.M.V., Nasseri K., Norman P., O’Donnell M., Omer S.B., Ortblad K., Osborne R., Ozgediz D., Pahari B., Pandian J.D., Rivero A.P., Padilla R.P., Perez-Ruiz F., Perico N., Phillips D., Pierce K., Pope C.A., Porrini E., Pourmalek F., Raju M., Ranganathan D., Rehm J.T., Rein D.B., Remuzzi G., Rivara F.P., Roberts T., De León F.R., Rosenfeld L.C., Rushton L., Sacco R.L., Salomon J.A., Sampson U., Sanman E., Schwebel D.C., Segui-Gomez M., Shepard D.S., Singh D., Singleton J., Sliwa K., Smith E., Steer A., Taylor J.A., Thomas B., Tleyjeh I.M., Towbin J.A., Truelsen T., Undurraga E.A., Venketasubramanian N., Vijayakumar L., Vos T., Wagner G.R., Wang M., Wang W., Watt K., Weinstock M.A., Weintraub R., Wilkinson J.D., Woolf A.D., Wulf S., Yeh P-H., Yip P., Zabetian A., Zheng Z-J., Lopez A.D., Murray C.J.L. (2012). Global and regional mortality from 235 causes of death for 20 age groups in 1990 and 2010: A systematic analysis for the global burden of disease study 2010.. Lancet.

[2] Mokdad A.A., Lopez A.D., Shahraz S., Lozano R., Mokdad A.H., Stanaway J., Murray C.J.L., Naghavi M. (2014). Liver cirrhosis mortality in 187 countries between 1980 and 2010: A systematic analysis.. BMC Med..

[3] Safaei A., Rezaei Tavirani M., Arefi Oskouei A., Zamanian Azodi M., Mohebbi S.R., Nikzamir A.R. (2016). Protein-protein interaction network analysis of cirrhosis liver disease.. Gastroenterol. Hepatol. Bed Bench.

[4] Ozsolak F., Milos P.M. (2011). RNA sequencing: Advances, challenges and opportunities.. Nat. Rev. Genet..

[5] Garber M., Grabherr M.G., Guttman M., Trapnell C. (2011). Computational methods for transcriptome annotation and quantification using RNA-seq.. Nat. Methods.

[6] Arakaki A.K., Skolnick J., McDonald J.F. (2008). Marker metabolites can be therapeutic targets as well.. Nature.

[7] Wang X., Zhang A., Han Y., Wang P., Sun H., Song G., Dong T., Yuan Y., Yuan X., Zhang M., Xie N., Zhang H., Dong H., Dong W. (2012). Urine metabolomics analysis for biomarker discovery and detection of jaundice syndrome in patients with liver disease.. Mol. Cell. Proteomics.

[8] Kivioja T., Vähärautio A., Karlsson K., Bonke M., Enge M., Linnarsson S., Taipale J. (2012). Counting absolute numbers of molecules using unique molecular identifiers.. Nat. Methods.

[9] Wang L., Feng Z., Wang X., Wang X., Zhang X. (2010). DEGseq: An R package for identifying differentially expressed genes from RNA-seq data.. Bioinformatics.

[10] Love M.I., Huber W., Anders S. (2014). Moderated estimation of fold change and dispersion for RNA-seq data with DESeq2.. Genome Biol..

[11] Abdi H. (2007). The bonferonni and šidák corrections for multiple comparisons..

[12] (2015). GBD 2013 Mortality and Causes of Death Collaborators. Global, regional, and national age–sex specific all-cause and cause-specific mortality for 240 causes of death, 1990–2013: A systematic analysis for the Global Burden of Disease Study 2013.. Lancet.

[13] Tsochatzis E.A., Bosch J., Burroughs A.K. (2014). Liver cirrhosis.. Lancet.

[14] Pan B.T., Johnstone R.M. (1983). Fate of the transferrin receptor during maturation of sheep reticulocytes in vitro: Selective externalization of the receptor.. Cell.

[15] Chaput N., Théry C. (2011). Exosomes: Immune properties and potential clinical implementations.. Semin. Immunopathol..

[16] Chen L., Charrier A., Zhou Y., Chen R., Yu B., Agarwal K., Tsukamoto H., Lee L.J., Paulaitis M.E., Brigstock D.R. (2014). Epigenetic regulation of connective tissue growth factor by MicroRNA-214 delivery in exosomes from mouse or human hepatic stellate cells.. Hepatology.

[17] Saito T., Harada K., Nakanuma Y. (2002). Granulomatous phlebitis of small hepatic vein.. J. Gastroenterol. Hepatol..

[18] Glass L.M., Su G.L.C. (2016). Metabolic Bone Disease in Primary Biliary Cirrhosis.. Gastroenterol. Clin. North Am..

[19] Guañabens N., Parés A., Mariñoso L., Brancós M.A., Piera C., Serrano S., Rivera F., Rodés J. (1990). Factors influencing the development of metabolic bone disease in primary biliary cirrhosis.. Am. J. Gastroenterol..

[20] Qamar A.A., Grace N.D., Groszmann R.J., Garcia-Tsao G., Bosch J., Burroughs A.K., Ripoll C., Maurer R., Planas R., Escorsell A., Garcia-Pagan J.C., Patch D., Matloff D.S., Makuch R., Rendon G. (2009). Incidence, prevalence, and clinical significance of abnormal hematologic indices in compensated cirrhosis.. Clin. Gastroenterol. Hepatol..

[21] Li B., Bailey A.S., Jiang S., Liu B., Goldman D.C., Fleming W.H. (2010). Endothelial cells mediate the regeneration of hematopoietic stem cells.. Stem Cell Res..

[22] Guillerey C., Harjunpää H., Carrié N., Kassem S., Teo T., Miles K., Krumeich S., Weulersse M., Cuisinier M., Stannard K., Yu Y., Minnie S.A., Hill G.R., Dougall W.C., Avet-Loiseau H., Teng M.W.L., Nakamura K., Martinet L., Smyth M.J. (2018). TIGIT immune checkpoint blockade restores CD8+ T-cell immunity against multiple myeloma.. Blood.

[23] Baniyash M., Sade-Feldman M., Kanterman J. (2014). Chronic inflammation and cancer: Suppressing the suppressors.. Cancer Immunol. Immunother..

[24] Choi W.M., Ryu T., Lee J.H., Shim Y.R., Kim M.H., Kim H.H., Kim Y.E., Yang K., Kim K., Choi S.E., Kim W., Kim S.H., Eun H.S., Jeong W.I. (2021). Metabotropic Glutamate Receptor 5 in Natural Killer Cells Attenuates Liver Fibrosis by Exerting Cytotoxicity to Activated Stellate Cells.. Hepatology.

[25] Li S., Ma D., Zhang L., Li X., Deng C., Qin X., Zhang T., Wang L., Shi Q., Wang Q., Wu Q., Zhang X., Zhang F., Li Y. (2013). High levels of FCγR3A and PRF1 expression in peripheral blood mononuclear cells from patients with primary biliary cirrhosis.. Dig. Dis. Sci..

[26] Fang S.S., Guo J.C., Zhang J.H., Liu J.N., Hong S., Yu B., Gao Y., Hu S.P., Liu H.Z., Sun L., Zhao Y.A. (2020). P53‐related microRNA model for predicting the prognosis of hepatocellular carcinoma patients.. J. Cell. Physiol..

[27] Jeong S., Kim S.A., Ahn S.G. (2021). HOXC6-Mediated miR-188-5p Expression Induces Cell Migration through the Inhibition of the Tumor Suppressor FOXN2.. Int. J. Mol. Sci..

[28] Deng J., Li Y.Q., Liu Y., Li Q., Hu Y., Xu J.Q., Sun T.Y., Xie L.X. (2019). Exosomes derived from plasma of septic patients inhibit apoptosis of T lymphocytes by down-regulating bad via hsa-miR-7-5p.. Biochem. Biophys. Res. Commun..

[29] Wei D., Sun L., Feng W. (2021). hsa_circ_0058357 acts as a ceRNA to promote non small cell lung cancer progression via the hsa miR 24 3p/AVL9 axis.. Mol. Med. Rep..

[30] Wang D. (2019). Zhang, Q.; Li, F.; Wang, C.; Yang, C.; Yu, H. β-TrCP-mediated ubiquitination and degradation of Dlg5 regulates hepatocellular carcinoma cell proliferation.. Cancer Cell Int..

[31] Weber-Boyvat M., Zhong W., Yan D., Olkkonen V.M. (2013). Oxysterol-binding proteins: Functions in cell regulation beyond lipid metabolism.. Biochem. Pharmacol..

[32] Hancock W.W., Wang L., Ye Q., Han R., Lee I. (2003). Chemokines and their receptors as markers of allograft rejection and targets for immunosuppression.. Curr. Opin. Immunol..

[33] Heijne W.H.M., Lamers R.J.A.N., van Bladeren P.J., Groten J.P., van Nesselrooij J.H.J., van Ommen B. (2005). Profiles of metabolites and gene expression in rats with chemically induced hepatic necrosis.. Toxicol. Pathol..

[34] Yang J., Xu G., Zheng Y., Kong H., Pang T., Lv S., Yang Q. (2004). Diagnosis of liver cancer using HPLC-based metabonomics avoiding false-positive result from hepatitis and hepatocirrhosis diseases.. J. Chromatogr. B Analyt. Technol. Biomed. Life Sci..

[35] Nagana Gowda G.A., Shanaiah N., Cooper A., Maluccio M., Raftery D. (2009). Visualization of bile homeostasis using (1)H-NMR spectroscopy as a route for assessing liver cancer.. Lipids.

[36] Attili A.F., Angelico M., Cantafora A., Alvaro D., Capocaccia L. (1986). Bile acid-induced liver toxicity: Relation to the hydrophobic-hydrophilic balance of bile acids.. Med. Hypotheses.

[37] Wang S., Sheng F., Zou L., Xiao J., Li P. (2021). Hyperoside attenuates non-alcoholic fatty liver disease in rats via cholesterol metabolism and bile acid metabolism.. J. Adv. Res..

[38] Amor F., Vu Hong A., Corre G., Sanson M., Suel L., Blaie S., Servais L., Voit T., Richard I., Israeli D. (2021). Cholesterol metabolism is a potential therapeutic target in Duchenne muscular dystrophy.. J. Cachexia Sarcopenia Muscle.

[39] Li G., Huang M., Cai Y., Yang Y., Sun X., Ke Y. (2019). Circ‐U2AF1 promotes human glioma via derepressing neuro‐oncological ventral antigen 2 by sponging hsa‐miR‐7‐5p.. J. Cell. Physiol..

